# 

**Published:** 1887-08-06

**Authors:** 


					h
PRICE ONE PENNY. .
S No. 45, Vol. II.
Ttigust 6th, 1887.
uuu, AOOI .
'Ristered at tlie General Post Offlc?
as a Xevrspaper.]
Per Annnm, 6s. 6d.,post free.
Monthly Farts, 6d.; post free, 7}d.
Ditto per Ann., 7s. 6d. post free.
ijVvH
ffifttHg ft )|f ' ??fc^
^gry]
JjijBPJSlipj
IPl
[jgliiBiiil
jSgSSfCTSr
J ? m ?^T^.f,^ toywch. Ifnontfnl,
an JiwtitutioH, Tamils, an* ^ $arocf)taI journal of
gjospttals. agplums. k all agencies for tf)e Care of tfle jbtcfe, grttictsm & ffetos.

				

